# Performance and educational training of radiographers in lung nodule or mass detection: Retrospective comparison with different deep learning algorithms

**DOI:** 10.1097/MD.0000000000026270

**Published:** 2021-06-11

**Authors:** Pai-Hsueh Teng, Chia-Hao Liang, Yun Lin, Angel Alberich-Bayarri, Rafael López González, Pin-Wei Li, Yu-Hsin Weng, Yi-Ting Chen, Chih-Hsien Lin, Kang-Ju Chou, Yao-Shen Chen, Fu-Zong Wu

**Affiliations:** a Department of Radiology, Kaohsiung Veterans General Hospital; b Department of Medical Imaging and Radiology, Shu-Zen Junior College of Medicine and Management, Kaohsiung; c Department of Biomedical Imaging and Radiological Sciences, National Yang Ming Chiao Tung University; d Department of Radiology, School of Medicine, College of Medicine, Taipei Medical University; e Department of Radiology, Wan Fang Hospital, Taipei Medical University, Taipei, Taiwan; f Radiology Department, Hospital Universitarioy Polite’cnico La Fe and Biomedical Imaging Research Group (GIBI230); g QUIBIM SL, Valencia, Spain; h Faculty of Medicine, School of Medicine, i Institute of Clinical Medicine, National Yang Ming Chiao Tung University; i Institute of Clinical Medicine, National Yang Ming University, Taipei; j Department of Medical Education and Research, Kaohsiung Veterans General Hospital, Kaohsiung, Taiwan.

**Keywords:** chest radiograph, deep-learning diagnosis, diagnostic performance, radiographers

## Abstract

The aim of this investigation was to compare the diagnostic performance of radiographers and deep learning algorithms in pulmonary nodule/mass detection on chest radiograph.

A test set of 100 chest radiographs containing 53 cases with no pathology (normal) and 47 abnormal cases (pulmonary nodules/masses) independently interpreted by 6 trained radiographers and deep learning algorithems in a random order. The diagnostic performances of both deep learning algorithms and trained radiographers for pulmonary nodules/masses detection were compared.

QUIBIM Chest X-ray Classifier, a deep learning through mass algorithm that performs superiorly to practicing radiographers in the detection of pulmonary nodules/masses (AUC^Mass^: 0.916 vs AUC^Trained radiographer:^ 0.778, *P* < .001). In addition, heat-map algorithm could automatically detect and localize pulmonary nodules/masses in chest radiographs with high specificity.

In conclusion, the deep-learning based computer-aided diagnosis system through 4 algorithms could potentially assist trained radiographers by increasing the confidence and access to chest radiograph interpretation in the age of digital age with the growing demand of medical imaging usage and radiologist burnout.

## 1 Introduction

Chest radiography is the most common use of radiologic medical imaging examination for lung cancer diagnosis.^[1,2]^ Although low-dose CT for lung cancer screening has been widely used in recent years, chest radiography is still the most commonly used tool for finding lung nodules or masses, especially in symptomatic patients.^[3–6]^ The clinical use of diagnostic chest radiography has increased tremendously over the past decade. Therefore, physician chronic stress and burnout are an extremely important matter for radiologists.^[7–9]^ Past studies have introduced that radiographers assist in chest radiograph diagnosis in the country with shortage of radiologists or high medical imaging demand.^[10–14]^ These studies have demonstrated that chest radiography interpretation by trained radiographers is not inferior to experienced radiologists.^[10,13]^ Previous study demonstrated that QUIBIM (Valencia, Spain) has developed a chest radiography classification software using different algorithm's approach that offers a solution to detect pulmonary nodules or masses, which can help radiology departments become more efficient in chest radiography interpretation in clinical practice through 14 pathology-specific 19-layer convolutional neural networks.^[15]^ However, the performance of these algorithms has not been compared to that of practicing radiographers. In this work, we aimed to investigate the performance of different deep learning algorithms to automatically interpret chest radiographs for pulmonary nodules or masses detection and evaluated its performance against practicing radiographers.

## 2 Methods

### 2.1 Study design and flowchart

The institutional board of Kaohsiung Veterans General Hospital, Taiwan approved this study and waived the need for patient consent, as the study was a retrospective review of already acquired chest radiographs (No. VGHKS18-CT11–07).

**Figure 1:**
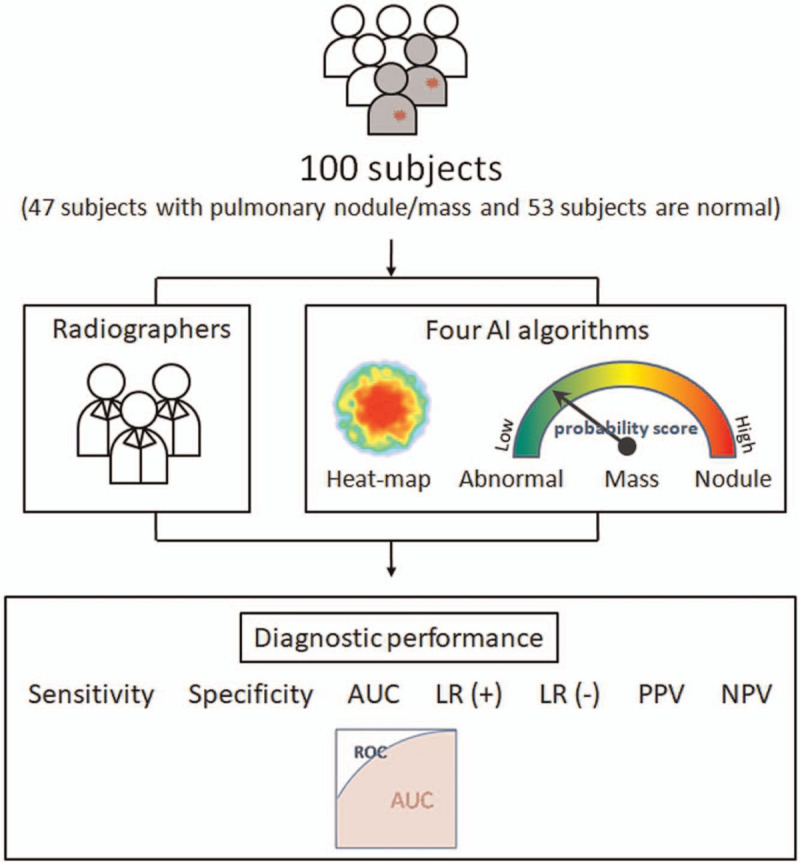
The flowchart of the comparison of the diagnostic performance of radiographers versus deep learning algorithms for pulmonary nodules/masses detection.

All selected 100 subjects with chest radiographs were de-identified, with patients’ names and identified number excluded from the details provided to radiographers and deep learning-based algorithms for imaging interpretation. The study population included 47 subjects with pulmonary nodules or masses and 53 subjects with normal chest radiographs as pointed out in the previous study.^[15]^ The presence of pulmonary nodules or masses was validated by chest CT exams. 100 study subjects are retrospectively evaluated by 6 radiographers and different deep learning algorithms independently. Before the actual reading sessions, all readers evaluated a training set of 5 cases. Readers are asked to interpret the chest radiographs according to standard steps. The first step is to determine whether there is a nodule or mass lesion in chest radiographs. The second step is to extract the region of interest if presence of a target lesion in the radiographs. The flowchart of the comparison of the diagnostic performance of radiographers versus deep learning algorithms for pulmonary nodules/masses detection is depicted in Figure 1.

### 2.2 Chest radiography interpretation process by radiographers

We compared deep learning algorithm's discriminative performance to the performance of 6 radiographers using the area under the receiver operating characteristic curve (AUC). The radiographers included 6 board-certified radiographers (average work experience 5.33 years, range 2–15 years). All participants self-reported their demographic information: age, gender, academic qualification, and employment status.

### 2.3 Chest radiography interpretation process by different deep learning algorithms

In our previous work, the deep learning algorithm software, called QUIBIM Precision could play a important role in the early detection of pulmonary nodules/masses on chest radiographs.^[15]^ The algorithms were modified and adopted by QUIBIM Precision and the software has been trained with ChestX-ray14 to estimate the probability of the presence of the 14 chest diseases using chest radiographs: atelectasis, cardiomegaly, pleural effusion, infiltration, mass, nodule, pneumonia, pneumothorax, consolidation, pulmonary edema, emphysema, fibrosis, pleural thickening, and hernia.^[16]^

As described previously, 4 different deep-learning algorithms for pulmonary nodules or masses detection were evaluated in this study to compare diagnostic performance between different deep learning algorithms, which included heat map algorithm, abnormal probability algorithm, nodule probability algorithm, and mass probability algorithm.^[15]^ Heat map is the ability to highlight the most abnormal region correctly on the heat map. Possibility score is the index value between 0 and 1 for abnormal probability algorithm, nodule probability algorithm, and mass probability algorithm. In this study, comparisons of the diagnostic performance of 4 different deep-learning algorithms for pulmonary nodules or masses detection to the trained radiographers are investigated.

### 2.4 Statistical analysis

All statistical analyses were performed with SPSS 17.0 for Windows (SPSS, Chicago, IL) and MedCalc 13.2.2.0 (MedCalc Software, Ostend, Belgium). Continuous variables are presented as mean ± standard deviation, and categorical variables as counts with proportions. Receiver operating characteristic (ROC) analysis was used to assess the performance of 4 deep learning algorithms and training radiographers, and to determine the optimal cut-off values of probability score, sensitivity, specificity, positive likelihood ratio (positive LR), negative likelihood ratio (negative LR), positive predictive value, negative predictive value and diagnostic accuracy were determined from the optimal threshold by the Youden index. In addition, we provide a comprehensive comparison of the 4 deep learning algorithms to trained radiographers. A comparison of the ROC curves was performed by using a method described by DeLong and colleagues.^[17]^ A *P* value of <.05 was considered significant. Generally, an AUC = 0.9–1.0 represents excellent, AUC = 0.8–0.9 good, AUC = 0.7–0.8 fair, and AUC = 0.6–0.7 poor discriminative ability according to the traditional academic points system.^[18,19]^

## 3 Results

Of the 100 subjects, 47 subjects were diagnosed with clinically significant pulmonary nodules/ masses and 53 subjects with normal finding for a prevalence rate of 47%, which were validated through chest CT images. Of the 100 study subjects, the ages of the subjects ranged from 18 to 88 years (mean 55.07 ± 13.80). For pulmonary nodule/mass anatomic lobar location, nodule size and radiographic nodule features are presented in Table 1.

**Table 1:** Baseline characteristics of 100 study subjects (subjects with pulmonary nodule/mass, n = 47; subjects without pulmonary nodule/mass, n = 53).

Mean age (yr)		55.07 ± 13.80 (18∼88)
Gender (%)	Male	54 (54%)
	Female	46 (46%)
Nodule size (cm) (%)		Mean 2.1 (0.7∼13.5)
	<1.5 cm	4 (9%)
	1.5–4 cm	20 (43%)
	>4 cm	23 (49%)
Nodule location (%)	Right upper lobe	15 (32%)
	Right middle lobe	2 (4%)
	Right lower lobe	5 (11%)
	Left upper lobe	15 (32%)
	Left lower lobe	10 (21%)
Radiologic nodule features (%)	Solid nodule	39 (83%)
	Part-solid nodule	8 (17%)

Six radiographers consented to participate in this study and completed the reading course. The trained radiographer's demographics are presented in Table 2. Of the 6 trained radiographers, the ages of the participants ranged from 28 to 45 years (mean = 31.7 years, rang 28–45 years-old). Most participants were female (5/6, 83.3%). Among these 6 participants, one of the participants is a master and the other 5 are bachelors. And most (66.7%) radiographers had less more than 5 years of working experience.

**Table 2:** Demographic characteristics of trained radiographers (n = 6).

Characteristic	Value	Frequency	Percentage
Mean Age (yr)	31.7 (28–45)	6	100%
Gender	Male	1	16.7%
	Female	5	83.3%
Education	Master's degree	1	16.7%
	Bachelor's degree	5	83.3%
Work experience in hospital	< 5 yr	4	66.7%
	5–10 yr	1	16.7%
	> 10 yr	1	16.7%

The diagnostic performance of four algorithms of QUIBIM Chest X-ray Classifier relative to trained radiographers has been summarized in Table 3, including the sensitivity, specificity, diagnostic accuracy, negative predictive value, positive predictive value, positive likelihood ratio (LR+), and negative LR (LR-) values. Among the diagnostic performance of four algorithms for pulmonary nodules/masses detection, the nodule probability algorithm was the most sensitive algorithm whereas the heat map algorithm was the most specific algorithm as previous described. In addition, the sensitivity of the performance of trained radiographers was 77.30% and the specificity was 78.30% for pulmonary nodules/masses detection. ROC curve analysis showed the only a fair predictive performance achieved with AUC of 0.778.

**Table 3:** Cut-off values and diagnostic performance from ROC curves in pulmonary nodule detection across different algorithms and trained radiographers.

	Cut-off	ROC	Sensitivity	Specificity	Positive LR	Negative LR	95% CI	PPV	NPV	Accuracy
Heat-map algorithm	(+)	0.682	38.30	98.11	20.3	0.63	0.643–0.719	95	64	0.70
Abnormal probability algorithm	>0.4116	0.810	74.47	83.02	4.39	0.31	0.776–0.841	78	78	0.78
Mass probability algorithm	>0.2884	0.916	76.60	90.57	8.12	0.26	0.891–0.937	86	81	0.83
Nodule probability algorithm	>0.2879	0.813	85.11	67.92	2.65	0.22	0.780–0.844	68	83	0.74
Trained radiographers	(+)	0.778	77.30	78.30	3.56	0.29	0.743–0.811	76	80	0.78

The comparisons of diagnostic performance between 4 algorithms relative to trained radiographers are summarized in Table 4. Compared with heat map algorithm, the radiographers achieved statistically significantly higher AUC performance on heat-map algorithm, with AUCs of 0.778 (95% CI 0.743–0.811). The mass algorithm achieved statistically significantly higher AUC performance on that of the radiographers, with AUCs of 0.916 (95% CI 0.891–0.937). For diagnostic performance of abnormal and nodule probability algorithms, there were no statistically significant differences in the AUCs compared to that of trained radiographers.

**Table 4:** Comparison of diagnostic performance between algorithms and radiographers for pulmonary nodule detection.

				
Algorithms	Algorithm (95% CI)	Radiographers (95% CI)	Difference between areas (Algorithm -Radiographers 95% CI)	Advantage
Heat-map algorithm	0.682 (0.643–0.719)	0.778 (0.743–0.811)	−0.096 (0.0561–0.136)	Radiographers
Abnormal probability algorithm	0.810 (0.776–0.841)	0.778 (0.743–0.811)	0.0321 (−0.0136–0.0777)	No difference
Mass probability algorithm	0.916 (0.891–0.937)	0.778 (0.743–0.811)	0.138 (0.100–0.176)	Mass algorithm
Nodule probability algorithm	0.813 (0.780–0.844)	0.778 (0.743–0.811)	0.0353 (−0.00911–0.0797)	No difference

## 4 Discussion

The results presented in this study demonstrate that QUIBIM Chest X-ray Classifier with the mass algorithm has been found to be superior in diagnostic performance for pulmonary nodules/masses detection than that of radiographers. In addition, the heat-map algorithm could automatically detect and localize pulmonary nodules/masses in chest radiographs with high specificity although this algorithm has inferior diagnostic performance compared to that of radiographers. To the authors’ knowledge, this is the first study to compare the diagnostic performance of AI deep learning four algorithms to that of radiographers for the detection of clinically significant pulmonary nodules/masses, which were validated by chest CT. The study has 3 major findings: first, mass algorithm had superior diagnostic accuracy with an AUC of 0.916 in comparison to that of trained radiographers. Second, the heat-map algorithm provides inferior performance as compared to that of trained radiographers. However, this algorithm has high specificity (low false-positive rate), which could help in assisting radiographers to make more accurate localization and diagnosis.

Third, trained radiographers had not been inferior diagnostic accuracy with that of abnormal and nodule probability algorithms by QUIBIM Chest X-ray Classifier.

These results indicate that we could use artificial intelligence and radiographer-assisted interpretation to assist radiologists in diagnosing and accelerating the process in an age with shortage of radiologists or high medical imaging demand.

We present QUIBIM Chest X-ray Classifier, a deep learning through the mass algorithm that performs superiorly to practicing radiographers in the detection of pulmonary nodules/masses in frontal-view chest radiographs. This study demonstrated that mass algorithm has been found to be superior in diagnostic performance for pulmonary nodules/masses detection than that of radiographers. In addition, the heat-map algorithm could automatically detect and localize pulmonary nodules/masses in chest radiographs with high specificity. Therefore, clinical integration of these algorithms could potentially assist trained radiographers by increasing the confidence and access to chest radiograph interpretation.^[15,20]^ Previous study has demonstrated that a rapid imaging processing time per case could help make clinical workflow more efficient.^[15]^ Radiographers can use real-time notification with high accurate score-based algorithm and high specific algorithm for lesion localization in a timely manner via integration with the PACS (picture archiving and communication system). Therefore, radiographers can act as an aid to pulmonary nodules/masses detection in a timely manner at the age of digital age with the growing demand of medical imaging usage and radiologist burnout.

There were 2 limitations to our study. First, this retrospective study aims to investigate the comparison of diagnostic performance of 4 algorithms relative to radiographers in pulmonary nodules/masses detection and localization. This study demonstrated the value of retrospective studies that mass algorithm has been found to be superior in diagnostic performance for pulmonary nodules/masses detection than that of radiographers. However further studies are needed to evaluate the clinical effect of combining artificial intelligence with radiographers to assist interpretation strategies in the real world. Second, this study only aimed to investigate the effectiveness of radiographers and artificial intelligence in interpreting pulmonary nodules/masses. However, there are many other important clinical diseases/findings that could be correctly diagnosed by chest radiograph such as pneumothorax and pleural effusion. Further studies are needed to evaluate the diagnostic performance of artificial intelligence relative to radiographers in the real-world practice.

## 5 Conclusion

In conclusion, we present QUIBIM Chest X-ray Classifier, a deep learning through the mass algorithm that performs superiorly to practicing radiographers in the detection of pulmonary nodules/masses in frontal-view chest radiographs. In addition, the heat-map algorithm could automatically detect and localize pulmonary nodules/masses in chest radiographs with high specificity. Therefore, clinical integration of these algorithms could potentially assist trained radiographers by increasing the confidence and access to chest radiograph interpretation in the age of digital age with the growing demand of medical imaging usage and radiologist burnout.

## Author contributions

**Conceptualization:** Pai-Hsueh Teng, Chia-Hao Liang, Fu-Zong Wu.

**Data curation:** Yun Lin, Fu-Zong Wu.

**Formal analysis:** Yun Lin, Fu-Zong Wu.

**Supervision:** Kang-Ju Chou, Yao-Shen Chen, Fu-Zong Wu.

**Writing – review & editing:** Pai-Hsueh Teng, Chia-Hao Liang, Angel Alberich-Bayarri, Rafael López González, Pin-Wei Li, Yu-Hsin Weng, Yi-Ting Chen, Chih-Hsien Lin, Kang-Ju Chou, Fu-Zong Wu.

## Correction

When originally published, the funding information was incomplete, “This study was supported by Grants from Kaohsiung Veterans General Hospital, VGHKS108–159, MOST108–2314-B-075B-008-, Taiwan, R.O.C.” This has since been corrected to “This study was supported by Grants from Kaohsiung Veterans General Hospital, VGHKS108 -159, MOST108 -2314-B-075B-008-, MOST 110-2314-B-075B-008, Taiwan, R.O.C.”
